# High-Anxious Individuals Show Increased Chronic Stress Burden, Decreased Protective Immunity, and Increased Cancer Progression in a Mouse Model of Squamous Cell Carcinoma

**DOI:** 10.1371/journal.pone.0033069

**Published:** 2012-04-25

**Authors:** Firdaus S. Dhabhar, Alison N. Saul, Tyson H. Holmes, Christine Daugherty, Eric Neri, Jean M. Tillie, Donna Kusewitt, Tatiana M. Oberyszyn

**Affiliations:** 1 Department of Psychiatry & Behavioral Sciences, Stanford University, Stanford, California, United States of America; 2 Stanford Cancer Institute, Stanford University, Stanford, California, United States of America; 3 Institute for Immunity, Transplantation, & Infection, Stanford University, Stanford, California, United States of America; 4 College of Medicine, The Ohio State University, Columbus, Ohio, United States of America; 5 Department of Carcinogenesis, The University of Texas M. D. Anderson Cancer Center, Smithville, Texas, United States of America; University of Medicine and Dentistry of New Jersey, United States of America

## Abstract

In spite of widespread anecdotal and scientific evidence much remains to be understood about the long-suspected connection between psychological factors and susceptibility to cancer. The skin is the most common site of cancer, accounting for nearly half of all cancers in the US, with approximately 2–3 million cases of non-melanoma cancers occurring each year worldwide. We hypothesized that a high-anxious, stress-prone behavioral phenotype would result in a higher chronic stress burden, lower protective-immunity, and increased progression of the immuno-responsive skin cancer, squamous cell carcinoma. SKH1 mice were phenotyped as high- or low-anxious at baseline, and subsequently exposed to ultraviolet-B light (1 minimal erythemal dose (MED), 3 times/week, 10-weeks). The significant strengths of this cancer model are that it uses a normal, immunocompetent, outbred strain, without surgery/injection of exogenous tumor cells/cell lines, and produces lesions that resemble human tumors. Tumors were counted weekly (primary outcome), and tissues collected during early and late phases of tumor development. Chemokine/cytokine gene-expression was quantified by PCR, tumor-infiltrating helper (Th), cytolytic (CTL), and regulatory (Treg) T cells by immunohistochemistry, lymph node T and B cells by flow cytometry, adrenal and plasma corticosterone and tissue vascular-endothelial-growth-factor (VEGF) by ELISA. High-anxious mice showed a higher tumor burden during all phases of tumor development. They also showed: higher corticosterone levels (indicating greater chronic stress burden), increased CCL22 expression and Treg infiltration (increased tumor-recruited immuno-suppression), lower CTACK/CCL27, IL-12, and IFN-γ gene-expression and lower numbers of tumor infiltrating Th and CTLs (suppressed protective immunity), and higher VEGF concentrations (increased tumor angiogenesis/invasion/metastasis). These results suggest that the deleterious effects of high trait anxiety could be: exacerbated by life-stressors, accentuated by the stress of cancer diagnosis/treatment, and mediate increased tumor progression and/or metastasis. Therefore, it may be beneficial to investigate the use of chemotherapy-compatible anxiolytic treatments immediately following cancer diagnosis, and during cancer treatment/survivorship.

## Introduction

Numerous studies have suggested that stress-prone personalities and coping styles, and factors like trait anxiety that contribute to increased stress, are associated with higher cancer incidence, progression or mortality [1]–[8]. However, many studies have failed to find an association between stress-related personality traits and cancer incidence and/or mortality [9]–[13]. Differences in the timing of personality assessment relative to cancer diagnosis, interactions between personality type, stressor severity, and related factors [14], [15], methodological heterogeneity among studies [16], and a focus on immunoresistant tumors in some studies [17], are thought to explain the equivocal nature of findings concerning personality factors and susceptibility to cancer. Importantly, cancer patients across diverse cultures consistently rank stress as a major contributing factor to their disease [18]–[21] and numerous studies have shown that long-term stress is related to increases in tumor incidence, progression, and metastasis [22]–[29] (for review see: [30]). In light of this background we investigated the association between anxiety-related behavioral phenotypes and cancer susceptibility under controlled laboratory conditions and in the context of an immuno-responsive cancer. Previous studies using the same tumor model described here, had shown that chronic stress increases susceptibility to squamous cell carcinoma (SCC) by suppressing protective immunity and increasing regulatory/suppressor T cells within the tumor microenvironment [25]. Anxiety may be defined as increased sensitivity and/or reactivity to actual and physically existent, or non-existent but perceived or anticipated threats/stressors. Because it is likely that a high-anxious behavioral phenotype can induce greater stress-reactivity and lead to an increased chronic stress load over time, we tested the *overall hypothesis that anxiety-related behavioral phenotypes measured at baseline (pre-cancer) would be associated with physiological indices of increased chronic stress, decreased protective immunity, and increased susceptibility to UV-induced SCC*.

The SCC model described here has several advantages: it involves a normal, immuno-competent, outbred mouse strain that has not been genetically manipulated, does not involve surgery or injection of exogenous tumor cells or cell lines, and allows investigation of the naturalistic time course of all phases of tumor development [25] with lesions and stages that closely resemble human SCC [31]–[33]. Importantly, the public health relevance of these studies is highlighted by the fact that the skin is the most common site of cancer in humans [34], with an estimated 2–3 million cases of non-melanoma skin cancers occurring each year worldwide [35]. SCC is the second most common type of cancer in the United States, with approximately 700,000 cases being diagnosed and resulting in approximately 2,500 deaths each year [36]. Furthermore, the ultraviolet B (UVB) component of sunlight (same wavelengths, 290–320 nm, used to induce SCC in the present model) is a complete carcinogen and is responsible for most non-melanoma skin cancers [37], [38]. Importantly, SCCs are immunogenic and can be eliminated by endogenous cell-mediated immune responses [39]–[41], however, UV exposure suppresses lymphocyte trafficking, and T and NK cell function [42], and inhibits anti-SCC immunity [43]–[45], all of which may enable SCC tumors to escape immune clearance [25], [46]. In light of the importance of anxiety being generated during the diagnosis and treatment of cancer, and the global health relevance and scientific value of the skin cancer model under study, here we investigate endocrine (physiological indices of chronic stress burden), immune (indices of protective versus suppressive immune function in the tumor microenvironment), and tumor growth-promoting (VEGF) mechanisms mediating the effects of high- versus low-anxious behavioral phenotypes on tumor emergence and progression.

## Results

### Classification of Mice into High- versus Low-anxious Phenotypes at Baseline

We used two established ethological tests of anxiety-related behavior, the elevated plus maze (EPM) and the light-dark arena (LDA), to determine the anxiety-related behavioral phenotype of each mouse [47]–[49] at baseline, i.e., before tumor induction. Two different tests were used because anxiety is a complex behavioral phenotype and we wanted to examine whether different dimensions of anxiety (that are likely to be evoked and quantified in different testing arenas) would be associated with specific aspects of tumor progression and/or tumor-relevant immune function. In addition, the open field arena (OFA) was used to determine the overall level of activity/locomotion as a control measure, to ensure that relationships observed using the EPM or LDA anxiety measures were not the result of inter-individual differences in overall activity levels [48], [50]. Mice were classified as high- or low- anxious on the basis of a median-split of the scores for each test. The resulting medians and group means are shown in Table 1. The difference between mice showing the high- versus low- anxious behavioral phenotype on each test was highly significant (p < =  0.0001) only when the above- versus below- median groups were compared for the test on which the median split was based, indicating independence among the behavioral measures.

**Table 1:** Classification of mice into low- versus high- anxious (EPM & LDA) or active (OFA) groups at baseline.

Behavioral Test	Measure	MEDIAN	MEAN (SEM) Below-Median Group	MEAN (SEM) Above-Median Group	*t*-test (*p)*
Elevated Plus Maze (EPM)	Time spent in closed arms (sec)	133	115 (3)	148 (2)	*
Light Dark Arena (LDA)	Time spent in dark (sec)	120	44 (8)	157 (4)	*
Open Field Arena (OFA)	Total distance travelled (cm)	456	311 (20)	683 (47)	*

* *p* < =  0.0001, *t*-test.

### High-anxious Mice Show Increased Tumor Emergence and Progression

**Figure 1:**
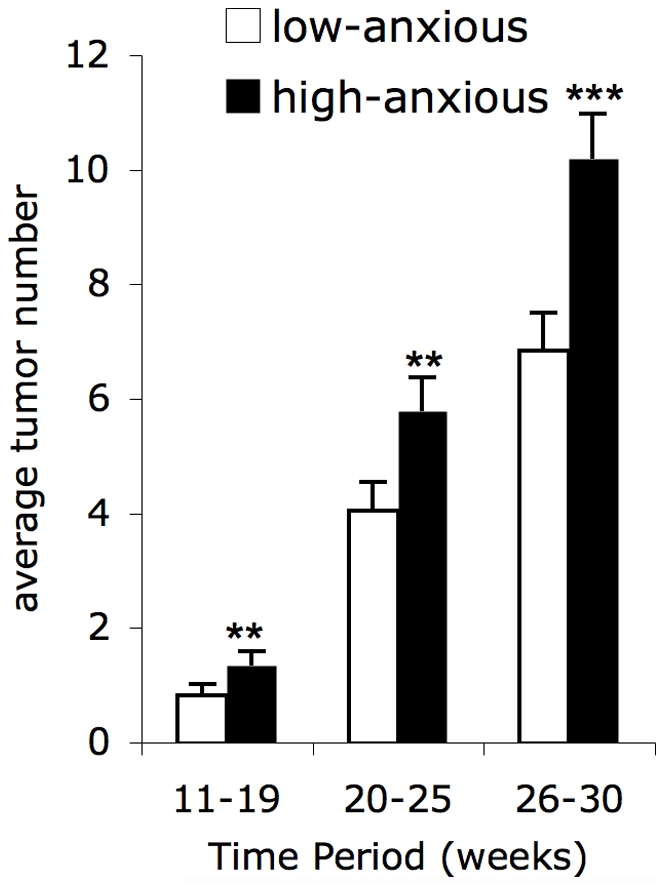
Effects of anxiety phenotype on tumor emergence and progression. Compared to low-anxious mice (white bars) high-anxious mice (black bars) showed a higher mean tumor count during all phases of tumor development: papilloma emergence and regression (∼weeks 11–19), transition to SCC (∼weeks 20–25), and SCC progression (∼week 26 onwards). Data are expressed as mean ± SEM. Statistically significant differences between phases are indicated (** *p* < 0.05; *** *p* < 0.01). Analysis employed a generalized linear model for a Poisson distribution on a logarithmic link function with allowance for overdispersion of the area data.

Mice that showed a high-anxious phenotype at baseline (as measured by the EPM) exhibited a greater tumor burden during all phases of tumor development (Fig. 1): papilloma emergence and regression (∼weeks 11–19), transition from papilloma to SCC (∼weeks 20–25), and SCC progression (∼week 26 onwards). Interestingly, no statistically significant differences in mean tumor counts were observed when mice were grouped into high- versus low- anxious phenotypes on the basis of their behavior in the LDA. Using total distance traveled in the OFA as a control measure for differences in overall activity levels between individual mice, we found no differences in tumor counts between high- versus low- locomotor activity phenotypes (p > 0.1). This suggested that the observed relationship between high-anxious phenotype and increased tumor burden is not the result of differences in overall locomotor activity but is related to anxiety. No statistically significant differences were observed in average tumor area, or time to 50% tumor incidence, when mice were grouped by high- versus low- anxious phenotypes, or by high- versus low- locomotor activity (p > 0.1). This suggested that while the high-anxious behavioral phenotype was associated with a higher count of tumors, no association was detected with tumor size or time to 50% tumor incidence.

The quantitative assessment of tumors described above was complimented with histopathological examination and identification of tissue characteristics of tumors at week 31. From the mice that were euthanized at week 31 we submitted one tumor from every animal that had a tumor with a diameter > 3 mm for histopathological classification. A total of eighteen tumors were classified. In mice characterized as high-anxious (HiAnx) in the EPM, one apparent tumor was reclassified as focal epidermal hyperplasia, a papilloma precursor lesion. Of the remaining tumors, four were papillomas, two were microinvasive SCC (miSCC), and two were fully invasive SCC. In mice characterized as low-anxious (LoAnx) in the EPM, five tumors were classified as papilloma, four as miSCC, and none as SCC. Mice characterized as HiAnx in the LDA had four papillomas, four miSCC, and two SCC. Among tumors sampled in mice characterized as LoAnx in the LDA, one was reclassified as focal epidermal hyperplasia, five were papillomas, two were miSCC, and none were SCC. Therefore, only mice characterized as HiAnx in either the EPM or the LDA had fully invasive SCC. Moreover, both groups of HiANx mice had a higher proportion of invasive miSCC or SCC compared to non-invasive papillomas (≥50% of tumors). Qualitatively, these findings suggest accelerated tumor progression in high-anxious individuals.

### Effects of High- versus Low-anxious Phenotypes on Chemokine and Cytokine Gene Expression in UV Exposed Skin and UV-induced Tumors

We examined the effects of high- versus low-anxious behavioral phenotypes on mean levels of gene expression of critical chemokines and cytokines that are known to have important protective (cutaneous T cell attracting chemokine (CTACK also known as CCL27), interleukin (IL)-12, interferon gamma (IFN-γ)) versus tumor-promoting (CCL22, IL-10, IL-4) effects in the context of SCC [25], [51]. Since overall biological function is ultimately influenced by relative proportions of counteracting factors, we also examined the effects of anxiety phenotype on the ratios of protective versus harmful chemokines (CTACK/CCL22) and cytokines ((IL-12+IFN-γ)/(IL-10 + IL-4)). To assess robustness of findings, effect size calculations (Cohen’s *d*), and multivariate regression analyses were performed in addition to *t*-tests. Therefore, *t*-test *p* values with effect size calculated as Cohen’s *d*, and/or regression *p* values with regression slope, are provided below for all analyses showing statistical significance (*p* < 0.05) or trend (*p* < 0.09). Importantly, for all comparisons where one of the two analyses, *t*-test or regression slope, showed a statistical trend, the remaining analysis showed statistical significance, the only exceptions being gene expression in UV exposed skin at week 17 for IFN-γ and at week 31 for the ratios of CTACK/CCL22 and (IL-12+IFN-γ)/(IL-4+IL-10), for which the *t*-test was not significant and the regression slopes only showed statistical trends.

**Figure 2:**
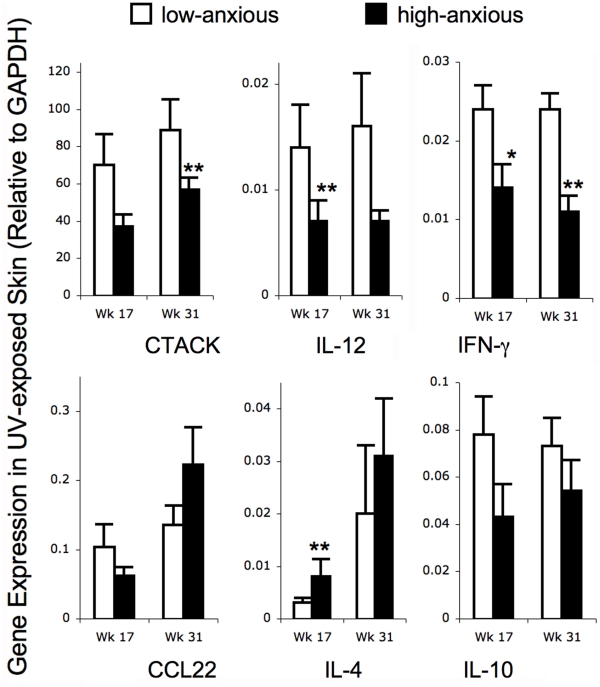
Effects of anxiety phenotype on chemokine and cytokine gene expression in UV-exposed skin. Gene expression in dorsal skin was measured by quantitative PCR at weeks 17 and 31 in low- (white bars) and high- (black bars) anxiety mice. mRNA levels of chemokines and cytokines known to be protective (CTACK, IL-12, and IFN-γ) or harmful (IL-4 and IL-10) in the context of SCC were quantified. Levels of mRNA expression normalized to glyceraldehyde-3-phosphate dehydrogenase (GAPDH) mRNA are shown. Data are expressed as mean ± SEM. Outcomes were optimally Box-Cox transformed prior to analysis. For each outcome, means were compared between groups using a two-sample *t*-test with Satterthwaite adjustment for unequal variances. Statistical trends (* *p* < 0.09) and significant differences (** *p* < 0.05) are indicated, and corresponding effect sizes are mentioned in the text. For all comparisons where the *t*-test showed a statistical trend, regression analysis showed statistical significance, the only exception being week 17 IFN-γ gene expression.

Gene expression was quantified in UV-exposed skin without visible lesions at weeks 17 and 31 in order to examine pre-cancerous changes (Fig. 2): At week 17, HiAnx mice showed lower levels of gene expression for CTACK (regression slope  =  –0.5, *p* < 0.05), IL-12 (*t*-test *p* < 0.05, effect size  =  0.9; regression slope  =  –0.5, *p* < 0.09) and IFN-γ (*t*-test *p* < 0.09, effect size  =  0.7) and higher gene expression for IL-4 (*t-*test *p* < 0.05, effect size  =  0.8; regression slope  =  0.5, *p* < 0.05) while CCL22 and IL-10 gene expression levels were not detectably different between the two groups (Fig. 2). At week 31, UV exposed skin of HiAnx mice showed lower levels of gene expression for CTACK (*t-*test *p* < 0.05, effect size  =  0.6; regression slope  =  –0.4, *p* < 0.01), IL-12 (regression slope  =  –0.4, *p* < 0.05) and IFN-γ (*t-*test *p* < 0.05, effect size  =  0.7; regression slope  =  –0.5, *p* < 0.01). CCL22, IL-4, and IL-10 gene expression at week 31 was not detectably different between HiAnx and LoAnx mice, however, HiAnx mice showed a lower ratio of CTACK/CCL22 (regression slope  =  –0.2 *p* < 0.09), and of protective (IL-12 + IFN-γ) to harmful (IL-10 + IL-4) cytokines (regression slope  =  –0.5, *p* < 0.09).

**Figure 3:**
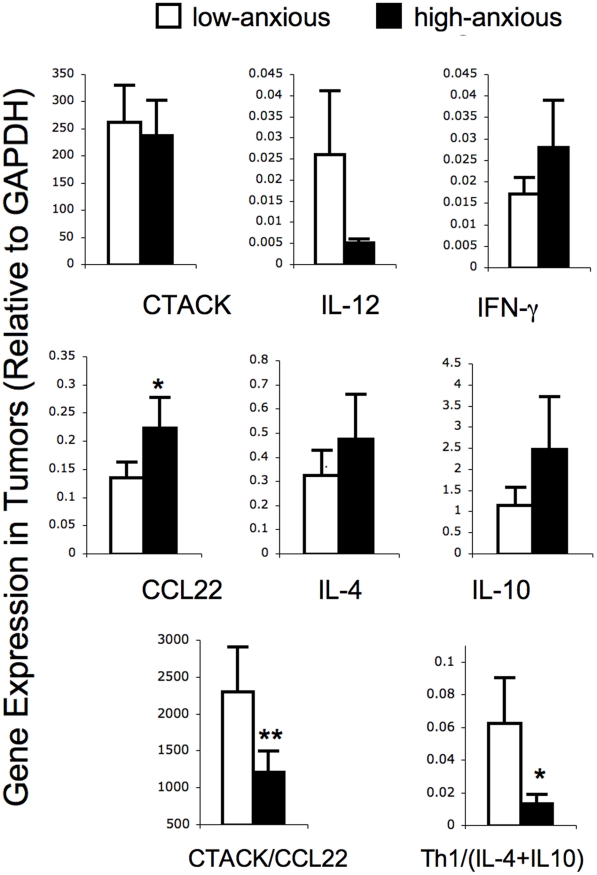
Effects of anxiety phenotype on chemokine and cytokine gene expression in tumors. Gene expression in tumors was measured by quantitative PCR at week 31 in low- (white bars) and high- (black bars) anxiety mice. mRNA levels of chemokines and cytokines known to be protective (CTACK, IL-12, and IFN-γ) or harmful (IL-4 and IL-10) in the context of SCC were quantified. Since overall biological function is ultimately influenced by relative proportions of counteracting factors, we also examined the effects of anxiety phenotype on the ratios of protective versus harmful factors: CTACK/CCL22 and (IL-12+IFN-γ)/(IL-10 + IL-4). Levels of mRNA expression normalized to glyceraldehyde-3-phosphate dehydrogenase (GAPDH) mRNA are shown. Data are expressed as mean ± SEM. Statistical trends (* *p* < 0.09) and significant differences (** *p* < 0.05) are indicated. Outcomes were optimally Box-Cox transformed prior to analysis. For each outcome, means were compared between groups using a two-sample *t*-test with Satterthwaite adjustment for unequal variances.

Gene expression was quantified in tumors at week 31 (Fig. 3): Tumors from HiAnx mice showed a higher levels of CCL22 gene expression (*t-*test *p* < 0.09, effect size  =  0.5; regression slope  =  0.4, *p* < 0.05), a lower ratio of CTACK/CCL22 (*t-*test *p* < 0.05, effect size  =  0.6; regression slope  =  –0.3 *p* < 0.05), and a lower ratio of protective (IL-12 + IFN-γ) to harmful (IL-10 + IL-4) cytokines (*t-*test *p* < 0.09, effect size  =  0.8; regression slope  =  –0.5, *p* < 0.05). The differences in gene expression between HiAnx and LoAnx mice described in this section were based on anxiety phenotype determined by the LDA, except for differences in IL-4 gene expression which were based on anxiety phenotype determined by the EPM.

### Tumors of High-anxious Mice Show Lower Numbers of Protective T cells and Higher Numbers of Regulatory/Suppressor T Cells

**Figure 4:**
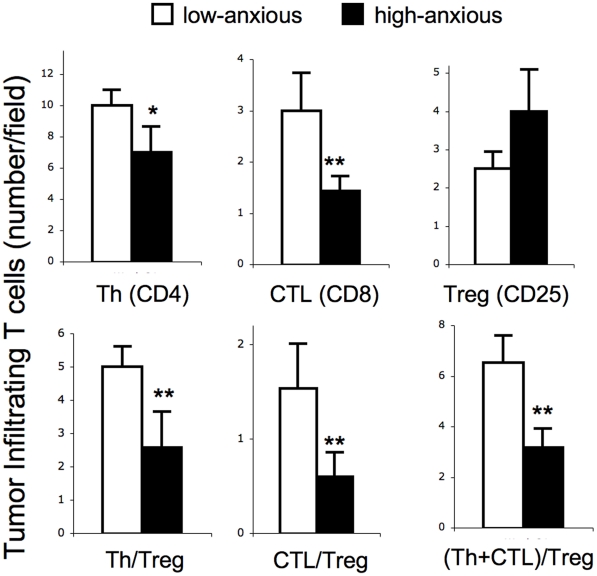
Effects of anxiety phenotype on tumor infiltrating helper (Th), cytolytic (CTL), and regulatory (Treg) T cells. Tumor infiltrating T cells were quantified in low- (white bars) and high- (black bars) anxiety mice at week 31. Numbers of tumor infiltrating cells that are known to be protective (Th and CTL) or harmful (Treg) in the context of SCC were quantified. Since overall biological function is ultimately influenced by relative proportions of counteracting cells, we also examined the effects of anxiety phenotype on the ratios of protective versus harmful factors: Th/Treg, CTL/Treg, and (Th + CTL)/Treg. The number of positive cells per standardized field was counted by a blinded observer. Five fields at 60x were analyzed per stained tumor section per mouse. Data are expressed as mean ± SEM. Statistical trends (* *p* < 0.09) and significant differences (** *p* < 0.05) are indicated. Outcomes were optimally Box-Cox transformed prior to analysis. For each outcome, means were compared between groups using a two-sample *t*-test with Satterthwaite adjustment for unequal variances.

**Figure 5:**
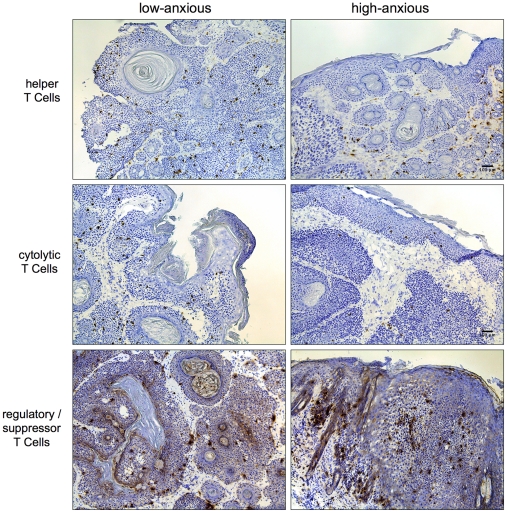
Photomicrographs showing the effects of anxiety phenotype on tumor infiltrating helper (Th), cytolytic (CTL), and regulatory (Treg) T cells. Immunohistochemical staining for CD4 (top row), CD8 (middle row), and CD25 (bottom row) was used to enumerate (counts shown in Fig. 4) tumor infiltrating T cells from low- (left column) and high- (right column) anxious mice at week 31. Scale bar denotes 100 µm.

We quantified the numbers of critical tumor infiltrating lymphocyte populations that are known to have important protective (helper and cytolytic T cells) or harmful (regulatory T cells) effects in the context of SCC in LoAnx and HiAnx mice (Fig. 4). Representative photomicrographs are also shown (Fig. 5). HiAnx mice as phenotyped on the basis of behavior in the LDA showed lower numbers of tumor infiltrating Th cells (*t-*test *p* < 0.07, effect size  =  0.6; regression slope  =  –0.5, *p* < 0.05) and CTLs (*t-*test *p* < 0.05, effect size  =  0.8), higher numbers of Tregs (regression slope  =  0.37, *p* < 0.09) and lower ratios of Th/Treg (*t-*test *p* < 0.05, effect size  =  1.0; regression slope  =  –0.5, *p* < 0.05), CTL/Treg (*t-*test *p* < 0.05, effect size  =  0.7; regression slope  =  –0.4, *p* < 0.09), and (Th+CTL)/Treg (*t-*test *p* < 0.05, effect size  =  1.1; regression slope  =  –0.6, *p* < 0.05). This suggests that the balance between immuno-protective versus immuno-regulatory/suppressive cells was shifted in favor of active Treg-mediated suppression of anti-tumor immunity in HiAnx mice.

### Sentinel Lymph Nodes of High-anxious Mice Show a Lower Proportion of CD4+ Helper T Cells and a Higher Proportion of B Cells

The relative percentages of Th (CD3+CD4+), CTL (CD3+CD8+) and B (B220+) cells in left and right brachial lymph nodes (2 lymph nodes per mouse) collected at week 31 are shown in Table 1. Brachial lymph nodes were examined because they are representative of sentinel lymph nodes that drain the dorsum, where a majority of the tumors were observed. Lymph nodes of high-anxious mice, phenotyped on the basis of their behavior in the LDA, showed a lower percentage of Th cells (*t-*test *p* < 0.05, effect size  =  0.7; regression slope  =  –0.5, *p* < 0.01) and higher percentage of B cells (*t-*test *p* < 0.01, effect size  =  0.8; regression slope  =  0.6, *p* < 0.01) compared to those of low-anxious mice. No detectable differences in percentages of CTLs were observed.

### High-anxious Mice Express Higher Levels of Total Adrenal and Peak Stress Corticosterone

We investigated the relationship between high- versus low-anxious phenotype and stress-reactivity of the hypothalamic-pituitary-adrenal (HPA) axis, measured by plasma corticosterone secretion, as a possible mechanism mediating the effects of anxiety phenotype on immune function and susceptibility to cancer. HiAnx mice, phenotyped on the basis of their behavior in the EPM (but not the LDA), showed higher total adrenal corticosterone levels than LoAnx mice (HiAnx  =  98.7 ± 11.5 µg, LoAnx  =  73.9 ± 9.4 µg, *t-*test *p* < 0.05, effect size  =  0.6; regression slope  =  0.3, *p* < 0.09). Resting state plasma corticosterone levels were not significantly different between HiAnx and LoAnx mice. However, when exposed to an acute stressor (0.5 h restraint), HiAnx mice, phenotyped on the basis of their behavior in the LDA (but not the EPM), also showed higher peak stress corticosterone levels compared to LoAnx mice (high-anxious  =  62.3 ± 6.3 µg/dL, low-anxious  =  47.6 ± 8.2 µg/dL, *t-*test *p* < 0.05, effect size  =  0.7). Taken together, these data indicate that HiAnx mice may be chronically exposed to higher levels of corticosterone, with higher peak stress corticosterone suggesting that high-anxious mice were likely to mount a greater stress response to moderate stressors such as cage changes or social interactions and with higher total adrenal corticosterone levels providing further evidence of a long-term or chronic increase in HPA axis activity.

### High-anxious Mice Express Higher Levels of Vascular Endothelial Growth Factor (VEGF)

Tissue VEGF has been used as a prognostic indicator for SCC [52], [53]. We investigated the relationship between high- versus low-anxious phenotype and VEGF concentrations within the tissue microenvironment of HiAnx versus LoAnx mice. HiAnx mice, phenotyped on the basis of their behavior in the LDA (but not the EPM), showed higher VEGF concentrations in skin lesions (diameter < 2 mm) that represent a transition from focal epidermal hyperplasia to true papilloma and are part of the spectrum of lesions leading ultimately to SCC [54], [55] (HiAnx  =  76.0 ± 16.7 pg/ml, LoAnx  =  45.1 ± 10.1 pg/ml, *t-*test *p* < 0.09, effect size  =  0.6). HiAnx mice also showed higher VEGF concentrations in UV-exposed skin where there were no visible lesions (HiAnx  = 29.9 ± 5.5 pg/ml, LoAnx  =  20.0 ± 2.7 pg/ml, *t-*test *p* < 0.09, effect size  =  0.6; regression slope  =  0.5, *p* < 0.05).

## Discussion

Numerous studies have shown that stress-prone personality and coping styles and factors like trait anxiety are associated with higher cancer incidence and/or increased mortality [1]–[8], however, other studies have failed to find associations between personality traits and cancer incidence and/or mortality [9]–[13]. Insightful reviews suggest that the equivocal nature of findings concerning personality factors and susceptibility to cancer are due to the timing of personality assessments relative to cancer diagnoses; interactions among personality type, stressor severity, and related factors [14], [15]; methodological heterogeneity among studies [16]; and a focus on immuno-resistant tumors in some studies [17]. The study described here investigated the association between baseline (pre-cancer) anxiety phenotype and cancer susceptibility under controlled laboratory conditions and in the context of an immuno-responsive tumor.

### Anxiety Phenotype and Tumor Burden

We tested the hypothesis that mice exhibiting a high-anxious behavioral phenotype at baseline would be more susceptible to UV-induced SCC compared to mice exhibiting a low-anxious phenotype. Supporting this hypothesis, we found that HiAnx mice exhibited a greater tumor burden during all phases of tumor development (Fig. 1): papilloma emergence and regression (∼weeks 11–19), transition from papilloma to SCC (∼weeks 20–25), and SCC progression (∼week 26 onwards). We found no further association between anxiety phenotype and time to tumor onset. This shows that a high-anxious phenotype does not hasten tumor incidence but is associated with increased tumor count and progression once tumors begin to emerge. These findings suggest that it may be beneficial, especially for high-anxious patients, to receive behavioral and/or chemotherapy-compatible pharmacological anxiolytic treatment soon after cancer diagnosis and during and following cancer treatment. These findings are in agreement with elegant studies in rats showing that a high-anxious, neophillic phenotype [56], or the anxiogenic and stress-inducing effects of social isolation [57], may contribute to increased susceptibility to mammary tumors.

### Chemokine and Cytokine Mediators

In order to identify immunological mechanisms that may mediate differences in tumor susceptibility between HiAnx and LoAnx mice, we examined gene expression of critical chemokines and cytokines known to exert protective versus harmful effects in the context of SCC. Factors likely to increase protective immunity against SCC were CTACK (also known as CCL27), a chemokine that is crucial for recruiting skin-homing T cells to sites of cutaneous immune activation [58]–[60], and IL-12 and IFN-γ, cytokines that are critical for the initiation and maintenance of cell-mediated [61], [62] and anti-tumor immunity [63]–[67]. Factors that are known to promote SCC progression and have been associated with poor prognosis included CCL22, a chemokine that attracts regulatory T cells (Tregs) that shut down the anti-tumor immune response [68]–[71], IL-10, an immuno-regulatory/suppressive cytokine [72]–[75], and IL-4, a prototypical Th2 cytokine [76]–[79]. Gene expression was quantified in skin that was devoid of detectable lesions in order to examine early changes in cutaneous immunity in the absence of tumors (Figure 2), as well as in tumors (Figure 3). Results showed that UV-exposed skin as well as tumors of HiAnx mice expressed lower levels of protective chemokines and cytokines than LoAnx mice. Since the biological actions of chemokines and cytokines often involve changes in the balance between counteracting factors, we also examined the effects of high- versus low-anxious phenotype on the ratio of protective to harmful factors. Tumors from HiAnx mice showed lower ratios of CTACK/CCL22 and (IL-12+IFN-γ)/(IL-10+IL-4). *Taken together, these data suggest that the chemokine balance in tumors of HiAnx mice is shifted towards CCL22, which attracts regulatory/suppressor T cells that suppress anti-tumor immunity, and away from CTACK, which may attract protective anti-tumor T cells*. *Correspondingly, the cytokine balance in HiAnx mice is shifted towards immuno-regulatory/suppressive (IL-10) and type-2 (IL-4) cytokines and away from Type-1 cytokines like IL-12 and IFN-*γ *that are known to protect against SCC*.

### Immune Cell Mediators

Given the differences in chemokine and cytokine gene expression described in the previous section, we quantified the effects of high- versus low-anxious phenotype on sizes of critical tumor infiltrating lymphocyte populations that are known to have important protective or harmful effects in the context of SCC (Fig. 4). Representative photomicrographs are also shown (Fig. 5). Cell types known to mount anti-tumor immune responses against SCC included CD4+ helper T cells (Th) and CD8+ cytolytic T cells (CTL) [40], [80]–[85]. The cell type known to favor tumor progression by suppressing anti-tumor immunity, was the CD25+ regulatory/suppressor T cell (Treg) [68]-[71], [86], [87]. Since the biological actions of these immune cell subpopulations often involve changes in the balance between competing cell types, we also examined the effects of high- versus low-anxious phenotype on the ratios of protective to harmful cells: Results showed lower numbers of infiltrating Th cells, higher numbers of Tregs, and lower ratios of CD4/CD25, CD8/CD25 and (CD4+CD8)/CD25 in tumors of HiAnx mice. *This suggests that the balance between immuno-protective versus immuno-regulatory/suppressive cells is shifted in favor of active suppression of protective anti-tumor immunity by Tregs in HiAnx mice.*

We also examined the distribution of immune cell subpopulations in brachial lymph nodes, the sentinel nodes that drain the dorsum where most tumors are observed. HiAnx mice showed a lower percentage and absolute number of Th cells and a higher percentage and absolute number of B cells. This shift in proportions of lymph node lymphocytes may be the result of the Type-1 to Type-2 cytokine shift observed in HiAnx mice. The increased tumor susceptibility of HiAnx mice may further result from the lower proportion of Th cells and a decrease in protective immunity in their sentinel lymph nodes. Importantly, the increase in lymph node B cell percentages and numbers in HiAnx mice may reflect an expansion of IL-10 secreting regulatory B cells (B regs) [88], [89]. While regulatory B cells need to be further investigated in the context of anxiety, UV exposure is thought to mediate immuno-suppression by activating regulatory B cells in sentinel lymph nodes [90] and inhibiting anti-tumor T cell responses in SCC [91]. Taken together, these findings suggest that in addition to increased regulatory T cells, increased regulatory B cell numbers and activity may also contribute to the higher IL-10 levels and mediate the decreased protective immunity and increased tumor burden observed in HiAnx mice.

### Differential Associations with High- versus Low-anxious Phenotype Measured in Different Ethological Arenas

We deployed two ethological tests for assessing anxiety-related behavior because anxiety is a complex behavioral phenotype and we wanted to determine whether different dimensions of anxiety (that are likely to be evoked and quantified in different testing arenas) are associated with specific aspects of tumor progression and/or tumor-related endocrine, angiogenic, or immune factors. Results showed that a high-anxious behavioral phenotype characterized in the EPM but not the LDA was associated with higher tumor counts, higher IL-4 gene expression in UV exposed skin, and higher total adrenal corticosterone concentrations. Interestingly, a high-anxious phenotype characterized in the LDA was associated with a shift from protective to harmful immune function, higher peak stress corticosterone concentrations, and higher tissue VEGF concentrations. While specific mechanisms mediating these differential relationships between different measures of anxiety and different biological parameters need to be investigated further, it is important to note that overall anxiety-related behaviors are thought to be the result of a balance between an animal’s need to avoid danger versus its motivation to explore novel environments to increase access to food and mates and that each of these measures of anxiety may tap into different dimensions of the complex behavioral phenotype. For example, anxiety-related quantitative trait loci (QTL) have been found on different mouse chromosomes [92]: a chromosome 15 QTL acts on avoidance behavior, and a chromosome 1 QTL acts on exploration [92]. The distinct physical characteristics of the EPM (elevated open “danger” zone) versus the LDA (flat illuminated “danger” zone) are likely to measure different dimensions of anxiety. These findings suggest that further research is necessary to identify the biological pathways and mechanisms that link specific dimensions of anxiety to tumor progression and/or tumor-related endocrine, angiogenic, or immune factors in order to provide targets for anxiety-ameliorating/eliminating pharmacological and/or behavioral interventions following cancer diagnosis and during cancer treatment. Importantly, these results show that whether defined on the basis of the EPM or the LDA, a high-anxious phenotype was associated with higher corticosterone concentrations (indicating increased chronic stress), suppressed protective immunity, higher VEGF (indicating greater tumor angiogenesis/metastasis) and significantly greater tumor burden. While much work remains to be done, the coherence of the overall pattern of findings that confirmed the *a priori* hypothesized biological changes and mechanisms: i.e., increased tumor burden, decreased chemokine, cytokine, and cellular indices of protective immunity, increased Treg-mediated suppression/tolerization of anti-tumor immunity, and increased corticosterone and VEGF levels, in HiAnx compared to LoAnx mice, and agreement with the literature regarding the effects of chronic stress on cancer [25], [26], also speaks to the usefulness and relevance of the approach used here.

### Role of Stress-reactivity, Chronic Stress, and Endocrine Factors

It is important to elucidate the neuroendocrine mechanisms mediating the effects of anxiety phenotype on immune function and/or tumor burden. Therefore, we hypothesized that a HiAnx behavioral phenotype results in greater stress-reactivity to daily life stressors that leads to increased chronic stress load over time. For example, for a given stressor (e.g., social altercations with cage mates, husbandry, cage changes, etc.), a HiAnx individual is likely to mount a larger physiological stress response compared to a LoAnx individual. Chronic stress has been shown to dysregulate physiological, endocrine, and immune function and to have numerous deleterious consequences that are thought to be mediated by chronic exposure to glucocorticoid and catecholamine stress hormones [93]–[96]. Specifically with respect to cancer, numerous studies have shown that long-term stress is related to increases in tumor incidence, progression, and metastasis [22]–[27], [97]–[99] (for review see: [30]). Chronic stress has also been shown to increase susceptibility to SCC by suppressing protective immunity and increasing regulatory T cells within the tumor microenvironment [25]. Since glucocorticoid hormones are important mediators of the effects of chronic stress [93], [94], [96], [100] we investigated the relationship between anxiety-related behavioral phenotype and resting-state total adrenal corticosterone and stress-induced plasma corticosterone, as mediators of the effects of anxiety phenotype on immune function and susceptibility to cancer. HiAnx mice showed higher total adrenal corticosterone and stress-induced peak corticosterone levels which suggests that HiAnx mice are chronically exposed to higher levels of corticosterone than LoAnx mice. The catecholamines, norepinephrine and epinephrine, are the other two members of the triad of principal stress hormones that together with cortisol/corticosterone mediate the effects of acute/short-term and chronic/long-term stress. Although we were not able to measure, and directly investigate the role of catecholamines, our results taken together with findings from other studies [26], [101]-[103], suggest that differences in systemic, tissue-associated, and/or tumor catecholamine levels are also likely to mediate the effects of moderate chronic stress arising from a high-anxious phenotype on suppressed immunity and increased susceptibility to SCC.

Several mechanisms have been proposed through which stress hormones can increase tumor burden. These include increased production of vascular endothelial growth factor (VEGF) [104], increased tumor metastasis [26], [105], decreased tumor anoikis [106] through activation of focal adhesion kinase [107], and dysregulation or suppression of immune function (for review see: [30], [99]). The role played by these mechanisms in mediating the effects of anxiety phenotype on tumor susceptibility merits further investigation.

### Role of VEGF

Tissue VEGF has been used as a prognostic indicator for SCC [52], [53] and VEGF has also been shown to be an important mediator of chronic stress-induced enhancement of tumor growth [26]. We did not have a sufficient number of tumors for VEGF analysis because tumors were used for histopathology, gene expression analysis and immunohistochemistry. However, we show here that HiAnx mice expressed higher levels of VEGF protein in SCC precursor lesions and also in UV-exposed areas of skin where no visible lesions were observed. This suggests that increased VEGF production within the tumor microenvironment of high-anxious mice may mediate the effects of anxiety-related behavioral phenotypes on SCC number and progression.

### Limitations and Future Directions

The limitations of this study are discussed and addressed as follows: First, some of the observed differences between HiAnx and LoAnx mice attained *p* values between 0.05 and 0.09 thus indicating statistical trends. This is likely due to power limitations that need to be addressed in future studies. However, to assess robustness of findings, effect size calculations (Cohen’s *d*), and multivariate regression analyses were performed in addition to *t*-tests. The estimated medium to large effect sizes (Cohen’s *d* shown for each comparison) speak to the possible statistical strength of the observed effects. Furthermore, with few exceptions, for all comparisons where one of the two analyses, *t*-test or regression slope, showed a statistical trend, the remaining analysis showed statistical significance. Importantly, the coherence of the overall pattern of findings that confirmed the *a priori* hypothesized biological changes and mechanisms: i.e., increased tumor burden, decreased chemokine, cytokine, and cellular indices of protective immunity, and increased corticosterone and VEGF levels, in HiAnx compared to LoAnx mice, and agreement with the literature regarding the effects of chronic stress on cancer [25], [26], suggests that the observed results were not random or due to false positive findings. Second, sample size limitations were likely to have been accentuated by the fact that these experiments used an outbred strain that is expected to show greater gene and gene-environment interaction driven variances than inbred strains. However, a significant advantage of using an outbred strain is that it provides a closer approximation of the genetic variation seen in human populations. Third, while the present study focused on the role of the HPA axis, the role of catecholamine hormones in mediating the effects of high-anxious phenotypes on increased tumor burden also needs to be examined. Fourth, while the present study focused on the role of the immune system in mediating the effects of anxiety phenotype on tumor susceptibility, other potential mechanisms (e.g. tumor anoikis [106]) remain to be investigated. Fifth, high-anxious mice are reported to exhibit significantly higher levels of oxidative stress in the brain and periphery [108] that suggests an oxidative stress related connection between HiAnx phenotype and tumor progression that also remains to be investigated. Sixth, the present study did not deliberately expose the mice to chronic stress and therefore did not examine the expected interaction between anxiety phenotype and chronic stress. Future studies need to test the hypothesis that the known deleterious effects of chronic stress on tumor incidence and/or progression [25], [26] will be exacerbated in HiAnx individuals.

### Clinical Implications

It is remarkable that an assessment of anxiety phenotype at baseline, before tumor induction, predicted increased chronic stress burden, lower protective immunity, increased tumor burden, and increased angiogenic factors in the tumor microenvironment, months later. While a high-anxious phenotype did not predict early tumor incidence, it predicted increased tumor burden once tumors began to emerge. In humans, trait anxiety has been associated with increased chronic stress before [109] and after [110], [111] cancer diagnosis/treatment. Therefore, it may be hypothesized that the effects of high trait anxiety may become particularly relevant after cancer diagnosis, could be accentuated by the stress of diagnosis and treatment, and may contribute to increased tumor emergence, progression, and/or metastasis through immune, stress-related endocrine, and other mechanisms. Indeed, several lines of evidence highlight the clinical importance of patient trait anxiety during cancer treatment and/or progression. High trait anxiety is associated with increased state anxiety before chemotherapy [110] and increased conditioned nausea and immuno-suppression during chemotherapy [111]. In agreement with the data presented here, Lutgendorf et al. have shown that higher levels of anxiety are associated with a Th1 to Th2 shift in lymphocytes obtained from peripheral blood, tumors, and ascites of ovarian cancer patients [112]. Pereira et al. have shown a positive association between anxiety and higher anti-heat shock protein 70 (HSP70) antibodies, indicating HSP70 over-expression related to tumorigenesis in patients with endometrial cancer [113]. Given that the SSC tumors studied here are known to be susceptible to immune attack, and given that the high-anxious phenotype is associated with lower anti-tumor immunity, our findings also have implications for tumor immunotherapy where the goal is to make immuno-resistant tumors responsive to elimination by the immune system. Taken together, these findings suggest that it may be beneficial to administer behavioral and/or chemotherapy-compatible pharmacological anxiolytic treatments to cancer patients (especially to high-anxious individuals) following cancer diagnosis and during cancer treatment, and to cancer survivors who report increased levels of anxiety. In addition to reducing the psychological and physiological burden of chronic stress, anxiolytic treatments are likely to conserve protective immunity which is not only important for tumor elimination in some cases, but also for overall immuno-protection against environmental pathogens and insults and for healing wounded/damaged tissues including those damaged by chemotherapy and radiation.

## Materials and Methods

### Mice

All mice were maintained and monitored and experiments were conducted according to protocol approved by the Institutional Laboratory Animal Care and Use Committee of The Ohio State University following guidelines established by the American Association for Accreditation of Laboratory Animal Care (AAALAC). Young adult (7–8 weeks) female SKH1 mice (Charles River, Wilmington, Massachusetts) were housed (5 per cage, except for two cages that had 4 mice each) in the AAALAC-accredited animal facilities of The Ohio State University. SKH1 hairless mice are widely used in studies involving skin cancer, and tumors closely resemble human SCC [31]–[33]. The animal room was maintained on a 12-hour light-dark cycle (lights on at 6 am). Mice were given food and water *ad libitum*.

### Experimental Design

All mice were phenotyped for anxiety-related behaviors at baseline following three weeks of acclimatization in the vivarium after arrival from the supplier. One week after behavioral phenotyping, all mice (n = 48) were exposed to UVB three times per week (∼10 minutes/session) for ten weeks as previously described [25], [51]. Mice were monitored for tumor development starting at week 11. Ten mice were euthanized (CO_2_ inhalation) during week 17 to elucidate effects on early stages of tumor formation and related measures of immune function. The rest of the mice (n = 38) were euthanized during week 31 to elucidate the effects of anxiety phenotype on SCC progression and related measures of immune factors, corticosterone and VEGF. Tissues were immediately dissected, frozen, and stored at –70°C. Tumors were either frozen for gene expression analysis, or fixed and processed for histological analysis (described below).

### Behavioral Phenotyping

We used two ethological tests of anxiety-related behaviors, elevated plus maze (EPM) and light-dark arena (LDA), to determine the anxiety-related behavioral phenotype of each mouse [47]–[49], and one test, open field arena (OFA), to determine the level of activity/locomotion [48], [50]. Mice were behaviorally phenotyped after allowing them to acclimatize for 3 weeks following arrival from the supplier. Behavioral testing was performed one week before the beginning of UV exposure. All animals were tested at the beginning of their active period. All testing equipment and software was from Accuscan Instruments (Columbus, Ohio). Anxiety Classification: The EPM and LDA tests quantify anxiety-related behaviors by creating conditions that tap into the intrinsic conflicts arising from the innate tendency of mice to explore (in order to increase the probability of finding food, water, and sex) and the innate fear/anxiety generated during exploration of well-lit, exposed, or elevated areas, which increases the probability of being attacked or killed by a predator or conspecific aggressor. These arenas have been used to measure trait anxiety in mice [114]–[116]. We used data from two ethological tests (EPM and LDA) because each test is thought to measure a different dimension of anxiety [92]. From the EPM we used the time spent in the closed arms: compared to mice with a low-anxious (LoAnx) phenotype, mice with a high-anxious (HiAnx) phenotype would spend more time in the closed, “protected,” arms versus the open, “exposed,” arms of the EPM. From the LDA we used time spent in the darkened area of the arena: HiAnx mice would spend more time in the dark, covered and “protected,” area of the arena, versus the lighted, open and “exposed,” area of the arena. Mice were classified as LoAnx versus HiAnx if respectively below versus above the median of their EPM and LDA scores that were measured at baseline. From the OFA we used total distance traveled as a control measure of the overall locomotor activity of each mouse. This was to ensure that any relationships observed using the EPM- or LDA-based anxiety phenotypes were not merely the result of inter-individual differences in overall activity levels. The resulting medians and group means are shown in Table 1.

### Tumor Counts and Measurements

None of the mice had SCC tumors at baseline. Mice were monitored for tumor development (emergence, number, and size) starting at week 11, when visible tumors first begin to appear. A tumor was defined as a mass having a diameter of at least 1 mm. Tumors were counted and measured weekly. The two longest measurements (mm) in perpendicular directions were made using a Cen-Tech digital caliper (Harbor Freight Tools, Camarillo, California) and were multiplied to obtain a representation of tumor area.

### Stress Test

At week 30, all mice were subjected to a short-term/acute stress test by placement in well-ventilated restrainers for 30 minutes. This procedure mimics stress that is largely psychological in nature because of the perception of confinement [117], [118] and activates the autonomic nervous system [119], the hypothalamic-pituitary-adrenal axis [120], [121], and adrenal steroid receptors in tissues throughout the body [120], [121]. Resting, and stress (30 min restraint) plasma corticosterone concentrations were quantified from ∼50 µl of blood that was collected into heparinized tubes from the retro-orbital sinus at each time point. Animals were restored to their home cages after completion of the stress test.

### Tumor Histology

Tumors were processed and histopathologic examination was performed on the largest tumor (diameter > 3 mm) from each animal as described previously [25], [51]. Tumors were classified by the team’s veterinary pathologist (DK).

### Measurement of Gene Expression

Gene expression was measured in dorsal skin collected and immediately frozen at weeks 17 and 31, and in tumors collected and frozen at week 31. CTACK, IL-12p40, IFN-γ, IL-4, IL-10, and CCL22 gene expression was quantified using real-time polymerase chain reaction (RT-PCR) as described previously [25], [51], [122].

### Preparation of Sentinel Lymph Node Cell Suspensions

Left and right brachial lymph nodes (2 per mouse) were placed in ice-cold Hanks Balanced Salt Solution (HBSS) (Invitrogen, Carlsbad, CA). A single cell suspension was prepared by squeezing between frosted ends of microscope glass slides (Fisher Scientific, Pittsburgh, PA), and filtering (70µm nylon) into HBSS. The cell suspension was then washed twice and re-suspended in HBSS. Cells were counted on a hematology analyzer (Beckman Coulter, Miami, FL) and stained for flow cytometric analysis as described below.

### Tumor Leukocyte Quantification by Immunohistochemistry

CD4+ and CD8+ and CD25+ T cells were quantified in tumors at week 31. Tumor specimens were sectioned, processed, and stained for CD4, CD8, and CD25 as described previously [25], [51]. Positive cells per standardized field were counted by a blinded observer. Five fields at 60x were analyzed per tumor section.

### Lymph Node Leukocyte Quantification by Flow Cytometry

Specific leukocyte subtypes were quantified in brachial lymph node cell suspensions using multicolor flow cytometry (FACSCalibur, Becton Dickinson, San Jose, CA). Cells were incubated with specific monoclonal antibodies for 25 min at room temperature, washed with PBS, and analyzed on the FACSCalibur. Directly conjugated rat anti-mouse CD3, CD4, CD8, and B220 (BD-Pharmingen, San Diego, CA) antibodies were used to label cells. Control samples matched for each fluorochrome and each antibody isotype were used to set negative staining criteria. Lymphocyte subtypes were identified as follows: helper T cells (CD3+CD4+), cytolytic T cells (CD3+CD8+), and B cells (B220+). Data were analyzed using CellQuest software (Becton Dickinson, San Jose, CA).

### Quantification of Plasma and Adrenal Corticosterone

Plasma corticosterone and total adrenal corticosterone were measured by colorimetric ELISA according to manufacturer’s instructions (Enzo Life Sciences, Ann Arbor, MI). Assay sensitivity was 0.03 µg/dL, and intra- and inter-assay CVs were 4% and 9% respectively.

### Quantification of Tissue VEGF

VEGF concentrations were measured in UV-exposed skin that had no visible lesions, as well as in skin lesions (diameter < 2 mm) that represent a transition from focal epidermal hyperplasia to true papilloma and are part of the spectrum of lesions leading ultimately to SCC [54], [55]. Each tissue was homogenized in lysis buffer per manufacturer instructions, and VEGF levels were quantified by a high-sensitivity sandwich immunoassay (Meso Scale Discovery, Gaithersburg, MD). Assay sensitivity was 1.4 pg/ml and the intra-assay CV was 7%.

**Table 2:** Percentages (%) and numbers (#) of lymphocytes in sentinel lymph nodes of low-anxious versus high-anxious mice.

IMMUNE CELL POPULATION	LOW-ANXIOUS mean (sem)	HIGH-ANXIOUS mean (sem)	t-test *p*	effectsize *d*	regression slope	regression *p*
Th (CD3+CD4+) %	49.2 (1.4)	44.9 (1.3)	**	0.7	–0.5	***
Th (CD3+CD4+) # (1000/µl)	12.4 (2.9)	7.3 (1.0)	*	0.7	–0.3	ns
CTL (CD3+CD8+) %	12.2 (1.2)	12.4 (1.4)	ns	ns	–0.04	ns
CTL (CD3+CD8+) # (1000/µl)	2.2 (0.5)	2.6 (0.3)	ns	ns	0.2	ns
B (B220+) %	34.0 (2.3)	42.5 (2.1)	***	0.8	0.6	***
B (B220+) # (1000/µl)	9.3 (2.4)	10.2 (1.8)	ns	ns	0.3	*

*** *p* < =  0.01; ** *p* < =  0.05; * *p* < =  0.09; ns  =  not statistically significant.

### Statistical Analyses

Figure 1: Tumor count and tumor area data were analyzed using a generalized linear model for a Poisson distribution on a logarithmic link function [123], with allowance for overdispersion of the area data. Analysis was performed separately for each *a priori* phase of tumor development: papilloma emergence and regression (∼weeks 11–19), transition from papilloma to SCC (∼weeks 20–25), and SCC progression (∼week 26 onwards). Within a period, count (or area) data were averaged and rounded to the nearest integer for each mouse. Rounded averages were multivariately regressed on binary variables for above vs. below median of each baseline anxiety variable plus an unordered categorical variable for cage (to account for possible clustering of responses within cages). Model assumptions were verified per McCullagh and Nelder [123]. Estimation employed least-squares means [124] to compare means of above-median vs. below-median groups separately for each baseline anxiety variable. Least squares means provide estimates of factor effects as if all factor-level combinations have equal sample sizes, even if sample sizes are unequal. This is especially important in experiments, where typically no reason exists to weight factor-level combinations differentially. Reported attained significance levels were one-sided *a priori*; and statistical significance, *p* < 0.05 or *p* < 0.01, is shown. Figures 2 , 3 , and 4 , Tables 1 & 2 , and corticosterone and VEGF results: Data were grouped as above vs. below median for each baseline behavior variable, for three separate two-group comparisons. Outcomes were optimally Box-Cox transformed [125] and compared between groups using a two-sample *t*-test with Satterthwaite adjustment for unequal variances. The corresponding effect size, *d*, a standardized measure of the strength of the difference between two groups, is also indicated with each *p* value. Application of Cohen’s standards for small, medium and large effect sizes indicated that most effect sizes fell in the “medium” to “large” range [126], even after adjustment for the tendency of small samples to overestimate effect size [127], [128]. To assess robustness of findings based on *t*-tests, we multivariately regressed each outcome on time spent in the darkened area of the arena and time spent in closed arms. Outcomes were Box-Cox transformed and then outcomes and regressors were centered and scaled [125], prior to analysis. Centering and scaling placed all regression coefficients on a common scale to aid interpretation. Estimated slopes farther from zero indicate stronger association. For *t*-tests and regression analyses, reported attained significance levels were one-sided *a priori*; and statistical significance, *p* < 0.05, or trend, *p* < 0.09, is shown. Analyses were performed in SAS(R) v.9.2 (SAS Institute, Cary, NC) and R v.2.9.1 (R Development Core Team 2009).
